# (3*E*,5*E*)-3,5-Bis(naphthalen-1-yl­methyl­idene)piperidin-4-one

**DOI:** 10.1107/S1600536812006307

**Published:** 2012-02-24

**Authors:** Yalda Kia, Hasnah Osman, Vikneswaran Murugaiyah, Suhana Arshad, Ibrahim Abdul Razak

**Affiliations:** a School of Chemical Sciences, Universiti Sains Malaysia, 11800 USM, Penang, Malaysia; b School of Pharmaceutical Sciences, Universiti Sains Malaysia, 11800 USM, Penang, Malaysia; c School of Physics, Universiti Sains Malaysia, 11800 USM, Penang, Malaysia

## Abstract

In the title compound, C_27_H_21_NO, the piperidine ring adopts a chair conformation. The mean plane through the piperidine ring makes dihedral angles of 49.27 (5) and 63.07 (5)° with the naphthalene ring systems. In the crystal, mol­ecules are linked into dimers *via* pairs of inter­molecular C—H⋯O inter­actions, generating ten-membered *R*_2_^2^(10) ring motifs. C—H⋯π inter­actions further stabilize the crystal structure.

## Related literature

For the biological activities of α,β-unsaturated ketones, see: Lee *et al.* (1971 ▶); Anke *et al.* (1981 ▶); Khodair *et al.* (1997 ▶); Murakami *et al.* (2002 ▶); El-Subbagh *et al.* (2000 ▶); El-Barbary *et al.* (1994 ▶); Dimmock *et al.* (1983 ▶). For ring conformations, see: Cremer & Pople (1975 ▶). For bond-length data, see: Allen *et al.* (1987 ▶). For a related structure, see: Basiri *et al.* (2011 ▶). For hydrogen-bond motifs, see: Bernstein *et al.* (1995 ▶). For experimental preparation, see: Das *et al.* (2007 ▶). For the stability of the temperature controller used for data collection, see: Cosier & Glazer (1986 ▶).
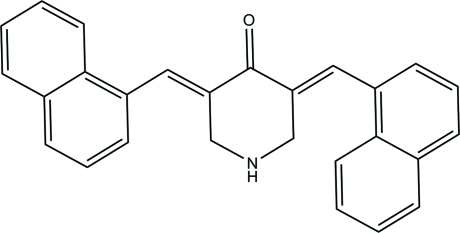


## Experimental

### 

#### Crystal data

C_27_H_21_NO
*M* *_r_* = 375.45
Monoclinic, 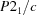

*a* = 9.4833 (2) Å
*b* = 10.0838 (2) Å
*c* = 20.3885 (4) Å
β = 101.513 (1)°
*V* = 1910.48 (7) Å^3^
*Z* = 4
Mo *K*α radiation
μ = 0.08 mm^−1^
*T* = 100 K
0.28 × 0.21 × 0.10 mm

#### Data collection

Bruker SMART APEXII CCD area-detector diffractometer
Absorption correction: multi-scan (*SADABS*; Bruker, 2009 ▶) *T*_min_ = 0.979, *T*_max_ = 0.992
21618 measured reflections
5644 independent reflections
4138 reflections with *I* > 2σ(*I*)
*R*_int_ = 0.037

#### Refinement

*R*[*F* ^2^ > 2σ(*F* ^2^)] = 0.051
*wR*(*F* ^2^) = 0.125
*S* = 1.02
5644 reflections
266 parameters
H atoms treated by a mixture of independent and constrained refinement
Δρ_max_ = 0.41 e Å^−3^
Δρ_min_ = −0.19 e Å^−3^

### 

Data collection: *APEX2* (Bruker, 2009 ▶); cell refinement: *SAINT* (Bruker, 2009 ▶); data reduction: *SAINT*; program(s) used to solve structure: *SHELXTL* (Sheldrick, 2008 ▶); program(s) used to refine structure: *SHELXTL*; molecular graphics: *SHELXTL*; software used to prepare material for publication: *SHELXTL* and *PLATON* (Spek, 2009 ▶).

## Supplementary Material

Crystal structure: contains datablock(s) global, I. DOI: 10.1107/S1600536812006307/rz2710sup1.cif

Structure factors: contains datablock(s) I. DOI: 10.1107/S1600536812006307/rz2710Isup2.hkl

Supplementary material file. DOI: 10.1107/S1600536812006307/rz2710Isup3.cml

Additional supplementary materials:  crystallographic information; 3D view; checkCIF report

## Figures and Tables

**Table 1:** Hydrogen-bond geometry (Å, °) *Cg*1 and *Cg*2 are the centroids of the C1–C6 and C1/C6–C10 rings, respectively.

*D*—H⋯*A*	*D*—H	H⋯*A*	*D*⋯*A*	*D*—H⋯*A*
C13—H13*B*⋯O1^i^	0.99	2.48	3.3758 (17)	150
C26—H26*A*⋯*Cg*1^ii^	0.95	2.92	3.7532 (15)	147
C25—H25*A*⋯*Cg*2^ii^	0.95	2.70	3.4923 (16)	141

Symmetry codes: (i) 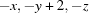; (ii) 
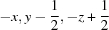
.

**Fig. 1.**
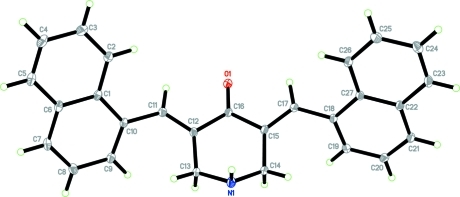
The molecular structure of the title compound, showing 30% probability displacement ellipsoids.

**Fig. 2.**
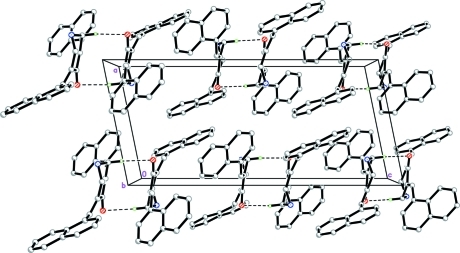
The crystal packing of the title compound viewed along the b axis. H atoms not involved in intermolecular hydrogen interactions (dashed lines) have been omitted for clarity.

## supplementary crystallographic information

### Comment

Reaction of aldehydes and ketones through Claisen-Schmidt condensation leads to α,β-unsaturated ketones which shown diverse biological activities such as cytotoxic (Lee *et al.*, 1971; Anke *et al.*, 1981; Khodair *et al.*, 1997), antitumor (Murakami *et al.*, 2002; El-Subbagh *et al.*, 2000) and antiviral activities (El-Barbary *et al.*, 1994). The conjugated O═CH—CH═CH2 system is the moiety which promotes the bioactivities in the title compound (Lee *et al.*, 1971). As reported by Dimmock *et al.* (1983), these class of compounds show cytotoxic activity without any subsidiary mutagenic and carcinogenic activities in human body.

The molecular structure is shown in Fig. 1. The piperidine ring (N1/C12–C16) adopts a chair conformation with puckering amplitude *Q* = 0.4308 (14) Å, θ = 40.80 (19)° and φ = 350.7 (3)° (Cremer & Pople, 1975). In addition, the mean plane through the piperidine ring makes dihedral angles of 49.27 (5) and 63.07 (5)° with the terminal naphthalene ring systems (C1–C10 and C18–C27), respectively. The bond lengths (Allen *et al.*, 1987) and angles are within normal ranges and comparable to those found in a the related structure (Basiri *et al.*, 2011).

In the crystal packing (Fig. 2), molecules are linked into dimers *via* pairs of intermolecular C13—H13B···O1 interactions (Table 1), generating ten-membered *R*^2^_2_(10) ring motifs (Bernstein *et al.*, 1995). The crystal structure is further stabilized by the intermolecular C26—H26A···*Cg1* and C25—H25A···*Cg2* (Table 1) interactions (*Cg*1 and *Cg2* are the centroids of the C1–C6 and C1/C6–C10 rings, respectively).

### Experimental

(3*E*,5*E*)-3,5-Bis(naphthalen-1-ylmethylene)piperidin-4-one was synthesized by the method described in the literature (Das *et al.*, 2007). Briefly, the title compound was prepared by dropwise addition of 1-naphtaldehyde (1 mmol) to a stirred mixture of 4-piperidone (1 mmol) and acetic acid (50 ml) in the presence of HCl (*g*) as catalyst at room temperature. After 24 h, a yellow precipitate formed and the completion of the reaction was monitored by TLC. The precipitate was filtered and washed with water. The pure solid was then recrystallized from ethanol to afford the title compound as yellow crystals.

### Refinement

The N-bound H atom was located in a difference Fourier map and refined freely [N–H = 0.928 (18) Å]. The remaining H atoms were positioned geometrically [C–H = 0.95–0.99 Å] and refined using a riding model with *U*_iso_(H) = 1.2 or 1.5 *U*_eq_(C).

### Crystal data

**Table:**

C_27_H_21_NO	*F*(000) = 792
*M**_r_* = 375.45	*D*_x_ = 1.305 Mg m^−^^3^
Monoclinic, *P*2_1_/*c*	Mo *K*α radiation, λ = 0.71073 Å
Hall symbol: -P 2ybc	Cell parameters from 5698 reflections
*a* = 9.4833 (2) Å	θ = 2.7–29.9°
*b* = 10.0838 (2) Å	µ = 0.08 mm^−^^1^
*c* = 20.3885 (4) Å	*T* = 100 K
β = 101.513 (1)°	Plate, yellow
*V* = 1910.48 (7) Å^3^	0.28 × 0.21 × 0.10 mm
*Z* = 4	

### Data collection

**Table:**

Bruker SMART APEXII CCD area-detector diffractometer	5644 independent reflections
Radiation source: fine-focus sealed tube	4138 reflections with *I* > 2σ(*I*)
Graphite monochromator	*R*_int_ = 0.037
φ and ω scans	θ_max_ = 30.2°, θ_min_ = 2.0°
Absorption correction: multi-scan (*SADABS*; Bruker, 2009)	*h* = −13→8
*T*_min_ = 0.979, *T*_max_ = 0.992	*k* = −13→14
21618 measured reflections	*l* = −28→28

### Refinement

**Table:**

Refinement on *F*^2^	Primary atom site location: structure-invariant direct methods
Least-squares matrix: full	Secondary atom site location: difference Fourier map
*R*[*F*^2^ > 2σ(*F*^2^)] = 0.051	Hydrogen site location: inferred from neighbouring sites
*wR*(*F*^2^) = 0.125	H atoms treated by a mixture of independent and constrained refinement
*S* = 1.02	*w* = 1/[σ^2^(*F*_o_^2^) + (0.0539*P*)^2^ + 0.7137*P*] where *P* = (*F*_o_^2^ + 2*F*_c_^2^)/3
5644 reflections	(Δ/σ)_max_ < 0.001
266 parameters	Δρ_max_ = 0.41 e Å^−^^3^
0 restraints	Δρ_min_ = −0.19 e Å^−^^3^

### Special details

**Table:**

Experimental. The crystal was placed in the cold stream of an Oxford Cryosystems Cobra open-flow nitrogen cryostat (Cosier & Glazer, 1986) operating at 100.0 (1) K.
Geometry. All e.s.d.'s (except the e.s.d. in the dihedral angle between two l.s. planes) are estimated using the full covariance matrix. The cell e.s.d.'s are taken into account individually in the estimation of e.s.d.'s in distances, angles and torsion angles; correlations between e.s.d.'s in cell parameters are only used when they are defined by crystal symmetry. An approximate (isotropic) treatment of cell e.s.d.'s is used for estimating e.s.d.'s involving l.s. planes.
Refinement. Refinement of *F*^2^ against ALL reflections. The weighted *R*-factor *wR* and goodness of fit *S* are based on *F*^2^, conventional *R*-factors *R* are based on *F*, with *F* set to zero for negative *F*^2^. The threshold expression of *F*^2^ > σ(*F*^2^) is used only for calculating *R*-factors(gt) *etc*. and is not relevant to the choice of reflections for refinement. *R*-factors based on *F*^2^ are statistically about twice as large as those based on *F*, and *R*- factors based on ALL data will be even larger.

### Fractional atomic coordinates and isotropic or equivalent isotropic displacement parameters (Å^2^)

**Table:**

	*x*	*y*	*z*	*U*_iso_*/*U*_eq_	
O1	0.20055 (10)	0.98893 (10)	0.10917 (5)	0.0220 (2)	
N1	−0.17776 (13)	0.77264 (12)	0.07711 (6)	0.0221 (3)	
C1	−0.17882 (15)	1.32180 (13)	0.12377 (7)	0.0184 (3)	
C2	−0.06600 (16)	1.34358 (14)	0.18022 (7)	0.0222 (3)	
H2A	0.0171	1.2887	0.1868	0.027*	
C3	−0.07534 (17)	1.44260 (15)	0.22535 (8)	0.0274 (3)	
H3A	0.0020	1.4567	0.2622	0.033*	
C4	−0.19864 (18)	1.52344 (16)	0.21750 (8)	0.0310 (4)	
H4A	−0.2053	1.5905	0.2495	0.037*	
C5	−0.30899 (17)	1.50529 (15)	0.16367 (8)	0.0274 (3)	
H5A	−0.3917	1.5604	0.1586	0.033*	
C6	−0.30191 (15)	1.40587 (14)	0.11546 (7)	0.0209 (3)	
C7	−0.41645 (16)	1.38733 (14)	0.05993 (7)	0.0233 (3)	
H7A	−0.4982	1.4438	0.0544	0.028*	
C8	−0.41112 (16)	1.28894 (14)	0.01388 (7)	0.0234 (3)	
H8A	−0.4880	1.2785	−0.0235	0.028*	
C9	−0.29140 (15)	1.20365 (14)	0.02233 (7)	0.0206 (3)	
H9A	−0.2890	1.1357	−0.0097	0.025*	
C10	−0.17705 (14)	1.21611 (13)	0.07607 (6)	0.0174 (3)	
C11	−0.05484 (15)	1.12471 (13)	0.08701 (6)	0.0181 (3)	
H11A	0.0365	1.1635	0.1039	0.022*	
C12	−0.05458 (14)	0.99261 (13)	0.07631 (6)	0.0168 (3)	
C13	−0.18459 (14)	0.90730 (13)	0.04956 (7)	0.0188 (3)	
H13A	−0.2712	0.9519	0.0593	0.023*	
H13B	−0.1962	0.9012	0.0003	0.023*	
C14	−0.04589 (15)	0.70382 (14)	0.07028 (7)	0.0217 (3)	
H14A	−0.0459	0.6913	0.0221	0.026*	
H14B	−0.0449	0.6149	0.0909	0.026*	
C15	0.08935 (14)	0.77746 (13)	0.10247 (6)	0.0173 (3)	
C16	0.08796 (14)	0.92523 (13)	0.09686 (6)	0.0171 (3)	
C17	0.20833 (15)	0.72198 (13)	0.13867 (6)	0.0185 (3)	
H17A	0.2798	0.7811	0.1613	0.022*	
C18	0.23999 (14)	0.57973 (13)	0.14716 (7)	0.0178 (3)	
C19	0.21653 (15)	0.49547 (14)	0.09261 (7)	0.0211 (3)	
H19A	0.1734	0.5291	0.0498	0.025*	
C20	0.25547 (15)	0.36044 (14)	0.09939 (7)	0.0236 (3)	
H20A	0.2389	0.3045	0.0611	0.028*	
C21	0.31695 (15)	0.30915 (14)	0.16068 (7)	0.0221 (3)	
H21A	0.3411	0.2177	0.1648	0.027*	
C22	0.34447 (14)	0.39198 (14)	0.21777 (7)	0.0189 (3)	
C23	0.41211 (15)	0.34198 (14)	0.28155 (7)	0.0228 (3)	
H23A	0.4373	0.2508	0.2862	0.027*	
C24	0.44152 (16)	0.42307 (15)	0.33632 (7)	0.0252 (3)	
H24A	0.4891	0.3886	0.3783	0.030*	
C25	0.40119 (15)	0.55807 (15)	0.33047 (7)	0.0238 (3)	
H25A	0.4207	0.6138	0.3688	0.029*	
C26	0.33416 (15)	0.60925 (14)	0.26995 (7)	0.0200 (3)	
H26A	0.3062	0.6999	0.2670	0.024*	
C27	0.30600 (14)	0.52860 (13)	0.21164 (7)	0.0171 (3)	
H1N1	−0.1808 (18)	0.7776 (18)	0.1223 (9)	0.030 (5)*	

### Atomic displacement parameters (Å^2^)

**Table:**

	*U*^11^	*U*^22^	*U*^33^	*U*^12^	*U*^13^	*U*^23^
O1	0.0211 (5)	0.0162 (5)	0.0274 (5)	0.0004 (4)	0.0013 (4)	−0.0002 (4)
N1	0.0224 (6)	0.0159 (6)	0.0273 (6)	0.0011 (5)	0.0037 (5)	0.0041 (5)
C1	0.0233 (7)	0.0136 (6)	0.0194 (6)	0.0007 (5)	0.0072 (5)	0.0015 (5)
C2	0.0239 (7)	0.0189 (7)	0.0244 (7)	0.0014 (6)	0.0064 (5)	−0.0019 (5)
C3	0.0323 (8)	0.0254 (8)	0.0243 (7)	−0.0021 (6)	0.0052 (6)	−0.0050 (6)
C4	0.0409 (9)	0.0239 (8)	0.0301 (8)	0.0032 (7)	0.0116 (7)	−0.0085 (6)
C5	0.0323 (8)	0.0211 (7)	0.0309 (8)	0.0076 (6)	0.0113 (6)	−0.0015 (6)
C6	0.0257 (7)	0.0151 (6)	0.0236 (7)	0.0026 (5)	0.0089 (5)	0.0023 (5)
C7	0.0254 (7)	0.0195 (7)	0.0257 (7)	0.0082 (6)	0.0071 (6)	0.0049 (5)
C8	0.0245 (7)	0.0229 (7)	0.0220 (7)	0.0048 (6)	0.0029 (5)	0.0036 (5)
C9	0.0264 (7)	0.0166 (7)	0.0191 (6)	0.0028 (5)	0.0053 (5)	0.0008 (5)
C10	0.0209 (6)	0.0128 (6)	0.0193 (6)	0.0014 (5)	0.0059 (5)	0.0013 (5)
C11	0.0203 (6)	0.0165 (6)	0.0176 (6)	0.0019 (5)	0.0039 (5)	0.0007 (5)
C12	0.0203 (6)	0.0148 (6)	0.0151 (6)	0.0026 (5)	0.0032 (5)	0.0014 (4)
C13	0.0200 (6)	0.0135 (6)	0.0222 (6)	0.0029 (5)	0.0024 (5)	0.0010 (5)
C14	0.0229 (7)	0.0139 (6)	0.0259 (7)	0.0020 (5)	−0.0010 (5)	0.0002 (5)
C15	0.0205 (6)	0.0128 (6)	0.0186 (6)	0.0016 (5)	0.0036 (5)	0.0000 (5)
C16	0.0216 (6)	0.0138 (6)	0.0155 (6)	0.0026 (5)	0.0028 (5)	−0.0005 (4)
C17	0.0206 (6)	0.0153 (6)	0.0190 (6)	0.0006 (5)	0.0022 (5)	−0.0002 (5)
C18	0.0166 (6)	0.0146 (6)	0.0217 (6)	0.0016 (5)	0.0026 (5)	0.0004 (5)
C19	0.0215 (7)	0.0186 (7)	0.0215 (7)	0.0026 (5)	0.0004 (5)	−0.0006 (5)
C20	0.0215 (7)	0.0184 (7)	0.0294 (7)	0.0016 (6)	0.0013 (6)	−0.0070 (5)
C21	0.0185 (7)	0.0137 (6)	0.0331 (7)	0.0012 (5)	0.0025 (5)	0.0001 (5)
C22	0.0146 (6)	0.0159 (6)	0.0260 (7)	0.0003 (5)	0.0036 (5)	0.0026 (5)
C23	0.0204 (7)	0.0176 (7)	0.0301 (7)	0.0015 (5)	0.0046 (6)	0.0078 (5)
C24	0.0226 (7)	0.0287 (8)	0.0233 (7)	0.0015 (6)	0.0023 (5)	0.0079 (6)
C25	0.0236 (7)	0.0273 (8)	0.0207 (7)	−0.0002 (6)	0.0049 (5)	0.0004 (5)
C26	0.0210 (7)	0.0161 (6)	0.0231 (7)	0.0017 (5)	0.0049 (5)	0.0002 (5)
C27	0.0152 (6)	0.0149 (6)	0.0211 (6)	0.0001 (5)	0.0032 (5)	0.0017 (5)

### Geometric parameters (Å, º)

**Table:**

O1—C16	1.2283 (16)	C13—H13A	0.9900
N1—C14	1.4610 (18)	C13—H13B	0.9900
N1—C13	1.4660 (17)	C14—C15	1.5136 (19)
N1—H1N1	0.928 (18)	C14—H14A	0.9900
C1—C2	1.4235 (19)	C14—H14B	0.9900
C1—C6	1.4251 (19)	C15—C17	1.3411 (18)
C1—C10	1.4451 (18)	C15—C16	1.4943 (18)
C2—C3	1.372 (2)	C17—C18	1.4686 (18)
C2—H2A	0.9500	C17—H17A	0.9500
C3—C4	1.408 (2)	C18—C19	1.3819 (18)
C3—H3A	0.9500	C18—C27	1.4344 (18)
C4—C5	1.369 (2)	C19—C20	1.410 (2)
C4—H4A	0.9500	C19—H19A	0.9500
C5—C6	1.415 (2)	C20—C21	1.371 (2)
C5—H5A	0.9500	C20—H20A	0.9500
C6—C7	1.416 (2)	C21—C22	1.414 (2)
C7—C8	1.374 (2)	C21—H21A	0.9500
C7—H7A	0.9500	C22—C23	1.4226 (19)
C8—C9	1.407 (2)	C22—C27	1.4242 (18)
C8—H8A	0.9500	C23—C24	1.367 (2)
C9—C10	1.3848 (19)	C23—H23A	0.9500
C9—H9A	0.9500	C24—C25	1.413 (2)
C10—C11	1.4626 (18)	C24—H24A	0.9500
C11—C12	1.3499 (18)	C25—C26	1.3711 (19)
C11—H11A	0.9500	C25—H25A	0.9500
C12—C16	1.4961 (18)	C26—C27	1.4210 (19)
C12—C13	1.5130 (18)	C26—H26A	0.9500
			
C14—N1—C13	112.12 (11)	N1—C14—C15	113.13 (11)
C14—N1—H1N1	108.5 (11)	N1—C14—H14A	109.0
C13—N1—H1N1	108.8 (11)	C15—C14—H14A	109.0
C2—C1—C6	118.03 (12)	N1—C14—H14B	109.0
C2—C1—C10	123.38 (12)	C15—C14—H14B	109.0
C6—C1—C10	118.55 (12)	H14A—C14—H14B	107.8
C3—C2—C1	121.04 (14)	C17—C15—C16	116.85 (12)
C3—C2—H2A	119.5	C17—C15—C14	125.52 (12)
C1—C2—H2A	119.5	C16—C15—C14	117.53 (11)
C2—C3—C4	120.54 (14)	O1—C16—C15	120.79 (12)
C2—C3—H3A	119.7	O1—C16—C12	121.31 (12)
C4—C3—H3A	119.7	C15—C16—C12	117.90 (12)
C5—C4—C3	119.88 (14)	C15—C17—C18	127.02 (12)
C5—C4—H4A	120.1	C15—C17—H17A	116.5
C3—C4—H4A	120.1	C18—C17—H17A	116.5
C4—C5—C6	121.12 (14)	C19—C18—C27	119.17 (12)
C4—C5—H5A	119.4	C19—C18—C17	120.63 (12)
C6—C5—H5A	119.4	C27—C18—C17	120.04 (12)
C5—C6—C7	120.91 (13)	C18—C19—C20	121.18 (13)
C5—C6—C1	119.36 (13)	C18—C19—H19A	119.4
C7—C6—C1	119.72 (13)	C20—C19—H19A	119.4
C8—C7—C6	120.98 (13)	C21—C20—C19	120.60 (13)
C8—C7—H7A	119.5	C21—C20—H20A	119.7
C6—C7—H7A	119.5	C19—C20—H20A	119.7
C7—C8—C9	119.77 (13)	C20—C21—C22	120.18 (13)
C7—C8—H8A	120.1	C20—C21—H21A	119.9
C9—C8—H8A	120.1	C22—C21—H21A	119.9
C10—C9—C8	121.78 (13)	C21—C22—C23	121.24 (13)
C10—C9—H9A	119.1	C21—C22—C27	119.81 (12)
C8—C9—H9A	119.1	C23—C22—C27	118.95 (13)
C9—C10—C1	119.13 (12)	C24—C23—C22	121.06 (13)
C9—C10—C11	122.35 (12)	C24—C23—H23A	119.5
C1—C10—C11	118.51 (12)	C22—C23—H23A	119.5
C12—C11—C10	128.62 (13)	C23—C24—C25	119.99 (13)
C12—C11—H11A	115.7	C23—C24—H24A	120.0
C10—C11—H11A	115.7	C25—C24—H24A	120.0
C11—C12—C16	115.70 (12)	C26—C25—C24	120.51 (14)
C11—C12—C13	126.27 (12)	C26—C25—H25A	119.7
C16—C12—C13	117.94 (11)	C24—C25—H25A	119.7
N1—C13—C12	114.71 (11)	C25—C26—C27	120.90 (13)
N1—C13—H13A	108.6	C25—C26—H26A	119.6
C12—C13—H13A	108.6	C27—C26—H26A	119.6
N1—C13—H13B	108.6	C26—C27—C22	118.56 (12)
C12—C13—H13B	108.6	C26—C27—C18	122.40 (12)
H13A—C13—H13B	107.6	C22—C27—C18	119.04 (12)
			
C6—C1—C2—C3	0.0 (2)	C14—C15—C16—O1	−164.86 (12)
C10—C1—C2—C3	−177.67 (14)	C17—C15—C16—C12	−161.14 (12)
C1—C2—C3—C4	1.3 (2)	C14—C15—C16—C12	15.56 (17)
C2—C3—C4—C5	−1.4 (2)	C11—C12—C16—O1	−14.65 (19)
C3—C4—C5—C6	0.2 (2)	C13—C12—C16—O1	168.69 (12)
C4—C5—C6—C7	179.89 (15)	C11—C12—C16—C15	164.93 (12)
C4—C5—C6—C1	1.1 (2)	C13—C12—C16—C15	−11.73 (17)
C2—C1—C6—C5	−1.2 (2)	C16—C15—C17—C18	−174.83 (13)
C10—C1—C6—C5	176.59 (13)	C14—C15—C17—C18	8.8 (2)
C2—C1—C6—C7	−179.99 (13)	C15—C17—C18—C19	46.2 (2)
C10—C1—C6—C7	−2.2 (2)	C15—C17—C18—C27	−138.58 (14)
C5—C6—C7—C8	−178.55 (14)	C27—C18—C19—C20	0.4 (2)
C1—C6—C7—C8	0.2 (2)	C17—C18—C19—C20	175.69 (13)
C6—C7—C8—C9	1.0 (2)	C18—C19—C20—C21	0.5 (2)
C7—C8—C9—C10	−0.3 (2)	C19—C20—C21—C22	−1.2 (2)
C8—C9—C10—C1	−1.7 (2)	C20—C21—C22—C23	−178.09 (13)
C8—C9—C10—C11	177.34 (13)	C20—C21—C22—C27	1.0 (2)
C2—C1—C10—C9	−179.42 (13)	C21—C22—C23—C24	178.48 (14)
C6—C1—C10—C9	2.94 (19)	C27—C22—C23—C24	−0.6 (2)
C2—C1—C10—C11	1.5 (2)	C22—C23—C24—C25	1.7 (2)
C6—C1—C10—C11	−176.17 (12)	C23—C24—C25—C26	−0.8 (2)
C9—C10—C11—C12	−37.3 (2)	C24—C25—C26—C27	−1.1 (2)
C1—C10—C11—C12	141.76 (14)	C25—C26—C27—C22	2.1 (2)
C10—C11—C12—C16	−176.09 (12)	C25—C26—C27—C18	−178.11 (13)
C10—C11—C12—C13	0.3 (2)	C21—C22—C27—C26	179.62 (13)
C14—N1—C13—C12	−53.18 (15)	C23—C22—C27—C26	−1.27 (19)
C11—C12—C13—N1	−145.86 (13)	C21—C22—C27—C18	−0.15 (19)
C16—C12—C13—N1	30.42 (17)	C23—C22—C27—C18	178.96 (12)
C13—N1—C14—C15	56.73 (15)	C19—C18—C27—C26	179.70 (13)
N1—C14—C15—C17	138.38 (14)	C17—C18—C27—C26	4.4 (2)
N1—C14—C15—C16	−38.00 (17)	C19—C18—C27—C22	−0.54 (19)
C17—C15—C16—O1	18.44 (19)	C17—C18—C27—C22	−175.85 (12)

### Hydrogen-bond geometry (Å, º)

Cg1 and Cg2 are the centroids of the C1–C6 and C1/C6–C10 rings, respectively.

**Table:**

*D*—H···*A*	*D*—H	H···*A*	*D*···*A*	*D*—H···*A*
C13—H13*B*···O1^i^	0.99	2.48	3.3758 (17)	150
C26—H26*A*···*Cg*1^ii^	0.95	2.92	3.7532 (15)	147
C25—H25*A*···*Cg*2^ii^	0.95	2.70	3.4923 (16)	141

Symmetry codes: (i) −*x*, −*y*+2, −*z*; (ii) −*x*, *y*−1/2, −*z*+1/2.
